# The long non-coding RNA BC200 (BCYRN1) is critical for cancer cell survival and proliferation

**DOI:** 10.1186/s12943-017-0679-7

**Published:** 2017-06-26

**Authors:** Evan P. Booy, Ewan KS McRae, Amit Koul, Francis Lin, Sean A. McKenna

**Affiliations:** 1 Department of Chemistry, University of Manitoba, Room 380 Parker Building, 144 Dysart Road, Winnipeg, MB R3T 2N2 Canada; 2 Department of Immunology, University of Manitoba, 750 McDermot Ave, Winnipeg, R3E 0T5 MB Canada; 3 Department of Physics & Astronomy, University of Manitoba, Allen Building, Winnipeg, R3T 2N2 MB Canada; 4 Department of Biochemistry & Medical Genetics, University of Manitoba, 745 Bannatyne Ave, Winnipeg, R3E 0J9 MB Canada

**Keywords:** BCYRN1, BC200, Long noncoding RNA (lncRNA), Cancer, Viability, Cell proliferation, Cell cycle, Apoptosis

## Abstract

**Background:**

BC200 is a long non-coding RNA expressed at high levels in the brain and elevated in a variety of tumour types. BC200 has a hypothesized role in translational regulation; however, to date the functional role of BC200 in both normal and diseased states remains poorly characterized.

**Methods:**

Detailed BC200 expression analyses were performed in tumor cell lines, primary and non-tumorigenic cultured breast and lung cells, and a panel of normal human tissues by quantitative real-time PCR and confirmed by northern blot. Subcellular fractionation was performed to assess BC200 distribution and efficient knock-down of BC200 was established using both locked nucleic acid (LNA) GapmeRs and conventional siRNAs. Cell viability following BC200 knockdown and overexpression was assessed by MTT assay and induction of apoptosis was monitored by Annexin V/PI staining and flow cytometry. Cell cycle arrest and synchronization were performed using serum withdrawal as well as the specific inhibitors Lovastatin, Thymidine, RO3306 and Nocodazole. Synchronization was monitored by fluorescent analysis of cellular DNA content by flow cytometry

**Results:**

BC200 expression was substantially upregulated in brain and elevated expression was also observed in testes, small intestine and ovary. Expression in cultured tumour cells was dramatically higher than corresponding normal tissue; however, expression in cultured primary cells was similar to that in immortalized and cancer cell lines. BC200 knockdown resulted in a dramatic loss of viability through growth arrest and induction of apoptosis that could be partially rescued by overexpression of wild-type BC200 but not an siRNA-resistant sequence mutant. A substantial decrease in BC200 expression was observed upon cell confluence or serum deprivation, as well as drug induced cell cycle arrest in G1 or G2 but not S- or M-phases. Upon release from cell cycle arrest, BC200 expression was recovered as cells entered S-phase, but did not follow a periodic expression pattern during synchronized progression through the cell cycle. This elevated expression was critical for the survival of proliferating cancerous and non-cancerous cells, but is dispensable upon senescence or cell cycle arrest.

**Conclusions:**

BC200 expression is elevated in proliferating cultured cells regardless of origin. In primary cells, expression is dramatically reduced upon cell cycle arrest by confluence, serum deprivation or chemical inhibition. The lethality of BC200 knockdown is restricted to actively proliferating cells, making it a promising therapeutic target for a broad spectrum of cancers.

**Electronic supplementary material:**

The online version of this article (doi:10.1186/s12943-017-0679-7) contains supplementary material, which is available to authorized users.

## Background

Brain Cytoplasmic RNA 1 (BCYRN1, BC200), herein referred to as BC200, is a 200 nucleotide RNA polymerase III transcript first identified by northern blot of primate brain cytoplasmic RNA extracts with a probe for the rat BC1 and BC2 RNAs [1]. The BC200 RNA can be divided into three distinct segments, the first consisting of 120 nucleotides that are homologous to the left monomer of Alu-J repetitive elements (Alu domain), the second a central 40 nucleotide adenosine rich stretch and the third, a unique 3′ region of 40 nucleotides that also possesses a continuous run of 12 cytosines [2–4]. Early studies suggest normal BC200 expression is confined to brain with only weak expression observed in testes and no detectable expression in other tissues [3]. Expression in brain is altered in the context of neurodegenerative disease and aging; however, results to date are contradictory in the context of Alzheimer’s Disease [5, 6]. Aberrant expression of BC200 is also reported in human tumours with substantially higher levels reported relative to matched normal tissue in cancers of the breast, lung, parotid gland, skin, stomach, esophagus, ovary, and cervix [7–11].

Despite a lack of sequence homology, primate-specific BC200 demonstrates a similar expression pattern as the mouse BC1 RNA with high neuronal levels and dendritic localization [1, 3]. In addition to a similar expression pattern, both BC1 and BC200 have been found to globally repress translation in both in vitro translation assays and when co-transfected with reporter mRNAs into HeLa cells [12–15]. In light of these results, BC200 is postulated to play a role in localized translational control in neuronal cells; however, specific mRNA targets of BC200 remain to be elucidated.

Several recent studies have begun to address the functional consequences of elevated BC200 expression in cancer. In the context of non-small-cell lung cancer, MYC-dependent BC200 upregulation is critical for cell migration and invasion [9]. Furthermore, a recent report has demonstrated estrogen regulated BC200 expression in breast cancer cell lines. The same study found that targeted deletion of BC200 in MCF-7 cells resulted in suppressed cell growth, altered morphology and elevated background levels of apoptosis. Furthermore, BC200 knock-out cells exhibited reduced tumour growth in murine xenograft models [10]. In contrast to the studies in breast and lung cancer, Wu et al. reported down-regulation of BC200 in ovarian cancer, with BC200 knock-down in ovarian cancer cell lines enhancing proliferation and having no impact on cell migration or invasion [16].

We have recently reported an interaction between BC200 and the RNA quadruplex helicase RHAU (DHX36) [2]. This study hypothesizes a role for RHAU in directing BC200 to target mRNA transcripts in a similar manner as has been proposed for the fragile X mental retardation protein (FMRP) [14, 17]. In the current study, we have sought to evaluate and clarify the role of BC200 in the context of cancer using a variety of cancerous cell lines and primary cell models. For the first time, we have performed detailed quantitative analyses of BC200 expression in a variety of human tissues, cancer cell lines and cultured primary cells that challenge the paradigm that BC200 is restricted to a neuronal and tumour expression pattern. We have confirmed that BC200 expression is largely restricted to the cytoplasm, and we have also established effective knock-down in a broad spectrum of cell types with both LNA GapmeRs and conventional siRNA. Our knock-down assays challenge reports that BC200 knockdown confers a survival advantage, as we clearly demonstrate growth arrest and induction of apoptosis in a broad spectrum of both cancer and normal primary cells. For the first time, we show that BC200 expression is greatly reduced in senescent and arrested cells and is elevated upon resumption of the cell cycle, suggesting the possibility of a distinct role for this long non-coding RNA outside of the nervous system.

## Methods

### Cell culture and reagents

The HEK293T cell line was a gift from Dr. Thomas Klonisch; the MCF-7, MDA-MB-231, SK-BR-3, T47D and HeLa cell lines were a gift from Dr. Spencer Gibson; the A549, SK-OV-3, IMR-90 and 16HBE cells were a gift from Dr. Peter Pelka and the MCF-10A cells were provided by Dr. Bob Varelas. The primary mammary epithelial cells (HMEC) were purchased from Thermo Fisher Scientific (Ottawa, Canada). MCF-10A and HMEC cells were grown in HuMEC media (Thermo Fisher Scientific), all other cells were cultured as published previously [2]. Synthetic RNA and DNA primers were purchased from Integrated DNA Technologies (Coralville, IA). LNA GapmeRs were purchased from Exiqon (Woburn, MA). siRNAs targeting BC200 and non-targeting controls were purchased from Qiagen (Toronto, CA), Integrated DNA Technologies and Thermo Fisher Scientific. Plasmids for BC200 overexpression were synthesized by Genscript Inc. (Piscataway, NJ). All standard laboratory chemicals and reagents were purchased from Thermo Fisher Scientific. DL-Mevalonic acid lactone, thymidine, cisplatin and etoposide were purchased from Sigma-Aldrich (Oakville, Canada). Lovastatin, nocodazole and RO3306 were purchased from Thermo Fisher Scientific. Rabbit anti-MYC (13987), mouse anti-Caspase-8 (9746), mouse anti-Caspase-2 (2224), and rabbit anti-Caspase-9 antibodies were purchased from Cell Signaling Technologies (Danvers, MA, USA) and the mouse anti-tubulin antibody was purchased from Sigma-Aldrich (T6074).

### RNA purification, RT-qPCR, and in-vitro transcription of BC200 RNA

RNA isolated from normal human tissue was purchased from Takara Bio (Mountain View, CA). RNA isolation from cultured cells was performed using the GeneJET RNA Purification kit as per the manufacturer’s protocol (Thermo Fisher Scientific). RNA was quantified using a Nanodrop 2000c spectrophotometer (Thermo Fisher Scientific). BC200 was in-vitro transcribed and purified by gel filtration chromatography as previously described [2, 18].

RT-qPCR analysis was performed using an Applied Biosystems StepOnePlus instrument (Thermo Fisher Scientific) with the iTaq Universal Green One-Step RT-qPCR kit (Bio-Rad, Mississauga, Canada). Reverse transcription and cycling parameters were carried out as per the manufacturer’s specifications (Bio-Rad iTaq Universal). To prepare a standard curve, serial dilutions of the BC200 RNA were made in RNase-free water containing 10 ng/μl murine carrier RNA. PCR efficiency was ~93% (Additional file 1: Figure S1). 25 ng of template RNA was used in all RT-qPCR reactions. RNA integrity was assessed following DNase digestion by electrophoresis and staining with the fluorescent dye SYBR Gold (Thermo Fisher Scientific). Reaction specificity was confirmed by melt-curve analysis as well as agarose gel electrophoresis of reaction products. No-template controls and no-RT controls amplified a non-specific product at approximately 37 cycles; this could not be avoided due to sequence constraints. The following primers were used: BC200-forward, ATAGCTTGAGCCCAGGAGTT; BC200-reverse, GCTTTGAGGGAAGTTACGCTTAT; BC200 siMUT-reverse, ATAAGACCGTAGCACACATCTAA; MALAT1-forward, GTTCTGATCCCGCTGCTATT; MALAT1-reverse, TCCTCAACACTCAGCCTTTATC; GRP94-forward, TGACTGAAGCACAGGAAGATG; GRP94-reverse, GCTACAAGGAAGGCGGAATAG; GAPDH-forward, ACCCACTCCTCCACCTTTG; GAPDH-reverse, CTCTTGTGCTCTTGCTGGG; MYC-forward, GCTGCTTAGACGCTGGATTT, MYC-reverse, GAGTCGTAGTCGAGGTCATAGTT.

### Subcellular fractionation

Sub-cellular fractionation was performed with the Thermo Scientific Subcellular Protein Fraction Kit for Cultured Cells (Thermo Fisher Scientific). 20 × 10^6^ cells were collected for fractionation with 10% set aside for total RNA isolation. RNA was extracted from 50 μl of each fraction with the GeneJet RNA Purification Kit following the RNA clean-up protocol (Thermo Fisher Scientific). RNA abundance was corrected for the RNA concentration and volume of each fraction relative to the total RNA isolation.

### Northern blotting

30 μg of RNA was combined with an equal volume of denaturing RNA load dye (95% deionized formamide, 0.025% sodium dodecyl sulfate (SDS), 0.025% bromophenol blue, 0.025% xylene cyanol FF, 0.5 mM ethylenediaminetetraacetic acid (EDTA)) and heated to 95 °C for five minutes. RNA was separated on 8% denaturing Tris-borate-EDTA-Urea (TBE-Urea) polyacrylamide gels followed by transfer to positively charged nylon membranes (Roche Life Science, Laval, Canada). After transfer, RNA was cross-linked to the membrane at 240 mJ/cm^2^ with a Spectrolinker XL-1000 UV cross-linker (Thermo Fisher Scientific). Membranes were subsequently incubated with shaking overnight at 60 °C with double-digoxigenin (DIG) LNA probes in Ultrahyb Oligo hybridization buffer (Thermo Fisher Scientific) containing 2× blocking reagent (Roche Life Science). Membranes were washed with 2X, 0.5X and 0.1X saline sodium citrate buffer (SSC) containing 0.1% SDS for 20 min at 60 °C. Blots were blocked for 30 min in 100 mM Maleic acid, 150 mM NaCl pH 7.5, with 2X blocking reagent. Primary (Mouse anti-Dig, Jackson ImmunoResearch, West Grove, PA) and secondary antibodies (Goat anti-mouse IGG, Thermo Fisher Scientific) were diluted in the same buffer and washes were performed with phosphate buffered saline containing 0.1% Tween 20 (PBS-T). Membranes were visualized by chemiluminescence (SuperSignal West Femto, Thermo Fisher Scientific). To visualize total RNA, a duplicate gel was stained with the fluorescent nucleic acid stain SYBR Gold (Thermo Fisher Scientific). Probe sequences are as follows: BC200-5′ probe, TCGAACTCCTGGGCTCAAGCTA; BC200-3′ probe, TTGAGGGAAGTTACGCTTATT.

### Plasmid, siRNA and LNA GapmeR transfection

Plasmid transfections were performed with Lipofectamine 3000 and siRNA/GapmeR transfections were performed with Lipofectamine RNAiMax according to the manufacturer’s protocols (Thermo Fisher Scientific). For overexpression, BC200 with 1747 bp upstream and 609 bp downstream flanking sequence was amplified from genomic DNA and cloned into the pUC57 vector (Genscript Inc). BC200 fused to the U6 promoter was synthesized by Genscript Inc. and cloned into the pUC57 vector. BC200-siMUT with nucleotides 163-185 scrambled was synthesized in the same manner. BC200 siRNA (targeted region) and GapmeR sequences are as follows: siRNA_1, CGCCUGUAAUCCCAGCUCUCA; siRNA_2, GAGACCUGCCUGGGCAAUAUA; siRNA_3, AGGCUAAGAGGCGGGAGGAUA; siRNA_4, AUAAGCGUAACUUCCCUCAAA (Qiagen); siRNA_5 GCUAAGAGGCGGGAGGAUATT (Life Technologies, Carlsbad CA); siRNA_6, CGUAACUUCCCUCAAAGCAACAACC (Integrated DNA Technologies); GapmeR_1 (Design ID 569710-1), TTGAGGGAAGTTACGC; GapmeR_2 (Design ID 569710-2), AGGGAAGTTACGCTTA; GapmeR_3, AACTCCTGGGCTCAA (Design ID 570780) (Exiqon). siRNA_6 and GapmeR_2 were employed for all experiments unless otherwise indicated. The following additional siRNA was used: MYC_1, AUCAUUGAGCCAAAUCUUAAAAAAA (Integrated DNA Technologies).

Co-transfection of plasmid DNA and siRNA or LNA GapmeRs was performed sequentially. Plasmid DNA was transfected using Turbofect (Thermo Fisher Scientific) as per the manufacturer’s protocol. 16 h following plasmid transfection cell culture media was changed and cells were incubated a further eight hours. Cells were then trypsinized, counted and reverse transfected with siRNA or LNA GapmeR at a cell density of 2 × 10^5^ cells/mL using Lipofectamine RNAiMax (Thermo Fisher Scientific).

### Viability and apoptosis assays

Cell viability was measured by the MTT assay as previously described [19]. Apoptosis assays were performed on a BD FACSCalibur flow cytometer (BD Biosciences, San Jose, CA) using the Dead Cell Apoptosis Kit with Annexin V Alex Fluor 488 and Propidium Iodide as per the manufacturer’s protocol (Thermo Fisher Scientific).

### Cell synchronization, cell cycle arrest and measurements of DNA content

MCF-10A cells were arrested at various stages of the cell cycle by treatment with 40 μM Lovastatin, 2 mM thymidine, 10 μM RO3306, and 0.1 μg/mL nocodazole as previously described [20]. For synchronization experiments, cells were harvested at two hour intervals for RNA extraction. Cells from each timepoint were fixed in ice cold 80% ethanol and stained in Tris-EDTA buffer containing 40 μg/mL propidium iodide (PI) and 40 μg/mL RNAse A. Cell cycle analysis was performed by measuring DNA content by PI fluorescence on the FL3 channel.

## Results

### Evaluation of BC200 expression in normal human tissue and tumour derived cell lines

As much of the previously published expression analyses of BC200 relied upon Northern blotting and in-situ hybridization methodologies [3, 7, 8], we sought to assess BC200 expression by performing direct quantification of RNA copy number by real-time quantitative PCR (RT-qPCR). Serial dilutions of purified in-vitro transcribed BC200 RNA generated a linear standard curve that spanned at least five orders of magnitude (Additional file 1: Figure S1a, b).

**Fig. 1:**
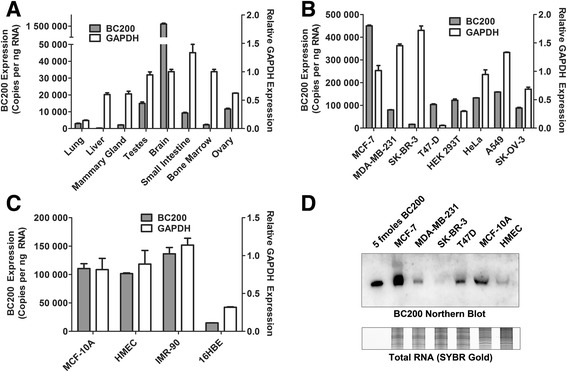
Analysis of BC200 expression in normal human tissue, cancer cell lines and primary cells. (**a**) BC200 expression was assessed by RT-qPCR in a panel of eight human tissues. Absolute BC200 quantification data is plotted on the left y-axis whereas relative GAPDH expression is plotted on the right y-axis. Data represents the mean of three replicate measurements +/− standard deviation. (**b**) As in (**a**), BC200 and GAPDH expression were assessed in a panel of eight immortalized cell lines. (**c**) As in (**a**), BC200 and GAPDH expression were assessed in a panel of four primary and non-tumorigenic cell lines. (**d**) BC200 expression was assessed by northern blot in a panel of six cell lines relative to 5 fmoles of in-vitro transcribed BC200 (*upper panel*). Total RNA was visualized on a duplicate gel by staining with SYBR Gold (*lower panel*)

BC200 expression was quantified in RNA extracted from eight different human tissues. In agreement with previous studies [1, 3], BC200 expression in brain was substantially higher than other tissues tested with an approximately 250-fold greater expression than the average of non-neuronal tissues. We also observed elevated expression in testes, ovary, and small intestine, whereas expression was considerably lower in lung, liver, mammary gland and bone marrow (Fig. 1a). BC200 expression was also measured in a panel of eight cell lines where an average of approximately 140,000 copies per ng RNA was observed. MCF-7 cells and SK-BR-3 cells demonstrated significant deviation from this average with MCF-7 cells expressing approximately 450,000 copies per ng and SK-BR-3 cells expressing approximately 16,000 copies per ng total RNA (Fig. 1b).

As BC200 expression is reported to be elevated in tumours of the breast and lung relative to normal tissue [3, 7, 9, 10], we also assessed BC200 expression in primary breast epithelial cells (HMEC) as well as the immortalized breast epithelial cell line MCF-10A, normal lung fibroblasts (IMR-90) and the bronchial epithelial cell line 16HBE (Fig. 1c). While 16HBE cells expressed significantly lower levels, expression in MCF-10A, HMEC and IMR-90 cells was comparable to that of the breast and lung cancer cell lines tested. Finally, northern blotting was employed as an complimentary method to confirm the relative abundance of BC200 in six of the tested cell lines (Fig. 1d).

### BC200 expression is primarily cytoplasmic

**Fig. 2:**
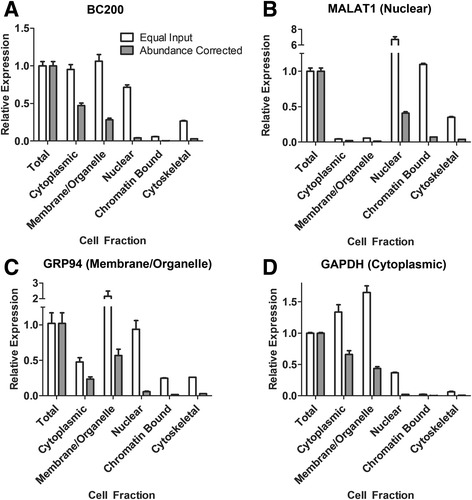
BC200 expression is primarily cytoplasmic. (**a**) Relative BC200 expression in indicated subcellular fractions was assessed by RT-qPCR. 25 ng of RNA was used as a template from each fraction (equal input). Relative RNA abundance was corrected according to the portion of the total cellular RNA present in each fraction (abundance corrected). (**b**) As in (a) with primers specific to MALAT1 (**c**) As in (**a**) with primers specific to GRP94. (**d**) As in (**a**) with primers specific to GAPDH

Qualitative in-situ hybridization analyses have suggested that BC200 expression is primarily cytoplasmic [3]. We sought to confirm cytoplasmic localization and quantify BC200 levels in various cellular compartments by cell fractionation and RT-qPCR. Purified RNA was extracted from soluble cytoplasmic, membrane-bound/organelle, nuclear soluble, chromatin bound and finally cytoskeletal fractions. MALAT1 was used as a nuclear RNA marker [21], GRP94 as a marker for the membrane/organelle fraction (endoplasmic reticulum) [22], and GAPDH for the soluble cytoplasmic fraction [23]. In agreement with previous observations, we found >95% of the total BC200 RNA to be in the cytoplasmic and membrane/organelle fraction (Fig. 2a), whereas the marker RNAs were enriched in their expected fractions (Fig. 2B-D).

### BC200 is efficiently knocked-down by LNA GapmeRs as well as siRNA

**Fig. 3:**
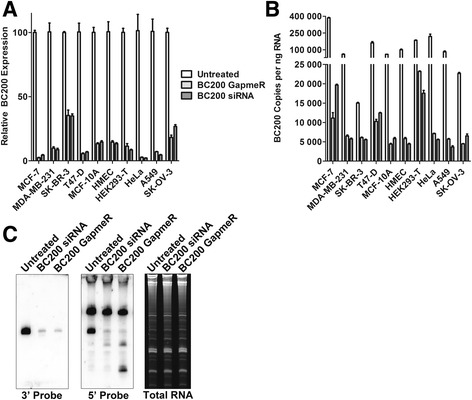
BC200 can be efficiently knocked down by siRNA and LNA GapmeR transfection. (**a**) Cells were transfected with a BC200 specific siRNA or GapmeR and harvested following 48-h. BC200 expression was assessed by RT-qPCR and normalized to the housekeeping gene GAPDH. (**b**) As in (**a**); however, absolute BC200 expression levels were assessed with a standard curve generated with the in-vitro transcribed RNA. (**c**) BC200 knock-down was confirmed in MCF-7 cells by northern blot with probes specific to a region in the 5′ end and 3′ end of the RNA. Total RNA was assessed by staining with SYBR Gold. The identity of the upper band in the middle panel is unknown

A previous report suggested that BC200 cannot be efficiently knocked down by siRNA [10], and indeed several other studies demonstrated sub-optimal knock-down efficiencies in the range of 60-80% [9, 16]. Sequence and structural constraints are a severely limiting factor to targeted knock-down of BC200; however, using Dicer substrate siRNAs as well as LNA GapmeRs targeting a unique sequence near the 3′ end of the RNA we were able to attain >95% knock-down in several cell lines (Fig. 3a). siRNAs targeting other regions within the RNA demonstrated poor knock-down efficiency; however, an LNA GapmeR targeting nucleotides 67-81 (GapmeR_3) was effective in several cell lines tested (Additional file 2: Figure S2A, B). Apparent variations in knock-down efficiencies between the cell lines tested were largely due to initial expression levels, as a reduction to approximately 5000 copies of BC200 per ng RNA appeared to be the lower limit of both the siRNA and GapmeR regardless of transfection conditions or cell line transfected (Fig. 3b). As we were unable to generate a specific and efficient qPCR primer set that did not overlap the RNA interference targeting sites of the RNA, a northern blot was performed to confirm that apparent knock-down efficiencies observed were not due to targeting oligonucleotides interfering with the qPCR assay (Fig. 3c).

### BC200 knock-down reduces viability in a broad spectrum of tumor cell lines

**Fig. 4:**
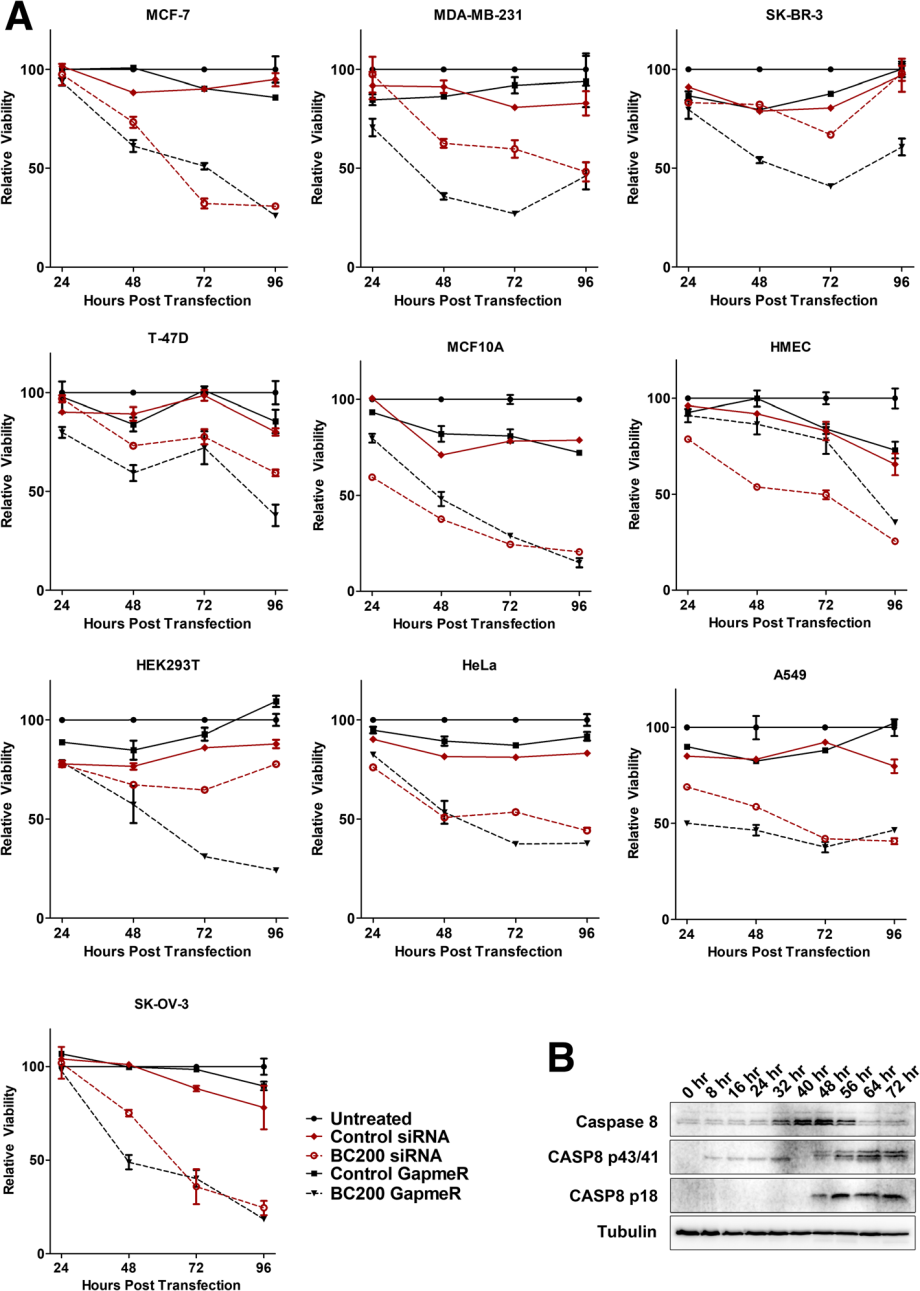
BC200 knock-down by siRNA or LNA GapmeR reduces cell viability. (**a**) BC200 was knocked down in each of the indicated cell lines and viability was assessed over the course of 96 h by MTT assay. Non-targeting siRNA and GapmeR controls were transfected in an identical manner to control for transfection mediated toxicity. Viability was measured relative to untreated cells at each timepoint. Data represents the mean of six biological replicates +/− standard error. (**b**) MCF-7 cells were collected every eight hours following BC200 siRNA transfection and protein was isolated and analyzed by SDS/PAGE and western blot. To assess caspase cleavage, blots were probed with a Caspase-8 antibody as well as a tubulin antibody as loading control

To date, the functional consequence of BC200 over-expression in tumor cells remains somewhat preliminary, with contradictory results reported in the literature. A study of non-small cell lung cancer cell lines reports a reduction in cell migration/invasion upon BC200 knock-down [9], and CRISPR/Cas9 knock-out of BC200 in MCF-7 cells showed reduced viability, altered morphology and susceptibility to apoptosis [10]. In contrast to these reports, a third study in ovarian cancer cells suggests BC200 knock-down has a beneficial impact on ovarian cancer cell line survival and chemo-resistance [16]. To gain clarity into the impact of BC200 knock-down on cell viability we performed BC200 knock-down time course assays and monitored cell viability by the MTT assay over 96 h in 10 distinct cell lines (Fig. 4a). All cell lines tested demonstrated a significant reduction in viability upon BC200 knock-down, by both siRNA and an LNA GapmeR as compared to appropriate controls. An LNA GapmeR targeting a region within the 5′ end of BC200 demonstrated a similar reduction in viability in cells in which knock-down was effective (Additional file 3: Figure S3). In a subset of cell lines, microscopic visualization of cells throughout the time-course was indicative of apoptotic morphology. To confirm induction of apoptosis, we performed a western blot for the initiating caspases 2, 8 and 9. While caspase 8 cleavage was observed within 48 h of BC200 siRNA transfection in MCF-7 cells (Fig. 4b), cleavage of caspase 2 and 9 was not observed (Additional file 4: Figure S4).

**Fig. 5:**
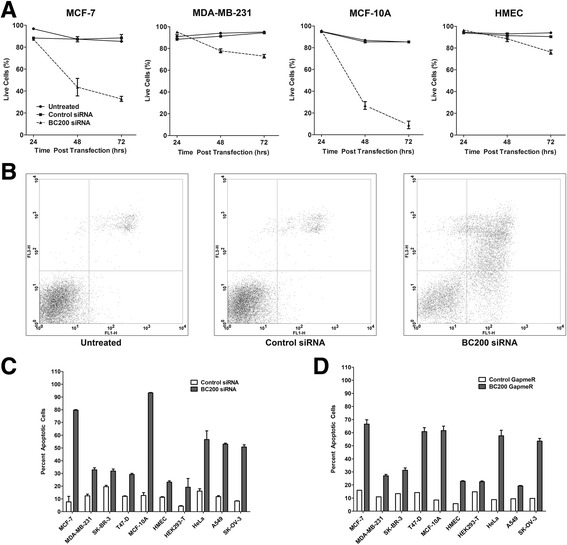
BC200 knock-down induces apoptosis. (**a**) BC200 was knocked down in the indicated cell lines by siRNA transfection. Cells were collected at the indicated time points and stained with Annexin V-Alexa Fluor 488 and propidium iodide to label apoptotic and dead cells. Data represents the fraction of unstained cells (lower left quadrant) at each timepoint measured in three biological replicates +/− standard error. (**b**) Dot plots of untreated, control siRNA and BC200 siRNA transfected MCF-7 cells at 72 h. Propidium iodide fluorescence is measured on the y-axis (FL3-H) and Annexin V-Alexa Fluor 488 is measured on the x-axis (FL1-H). (**c**) Apoptosis was assessed as in (**a**) following 96-h transfection with a BC200 targeting siRNA. (**d**) Apoptosis was assessed as in (**a**) following 96-h transfection with a BC200 targeting LNA GapmeR

To determine the contribution of programmed cell death to the loss of viability in all cell lines, apoptosis was measured over the course of 72-h by staining cells with Annexin V-Alexa Fluor 488 and propidium iodide (Fig. 5a, b). Phosphatidylserine exposure and membrane permeability were observed 48-h post BC200 siRNA transfection in MCF-7, MDA-MB-231 and MCF-10A cells whereas HMEC cells did not show significant induction of cell death until 72-h. In a similar manner, apoptosis was measured in all cells at 96 h. BC200 knock-down by both the siRNA and GapmeR resulted in a substantial increase in cell death as compared to appropriate controls in all cell types tested (Fig. 5c, d).

**Fig. 6:**
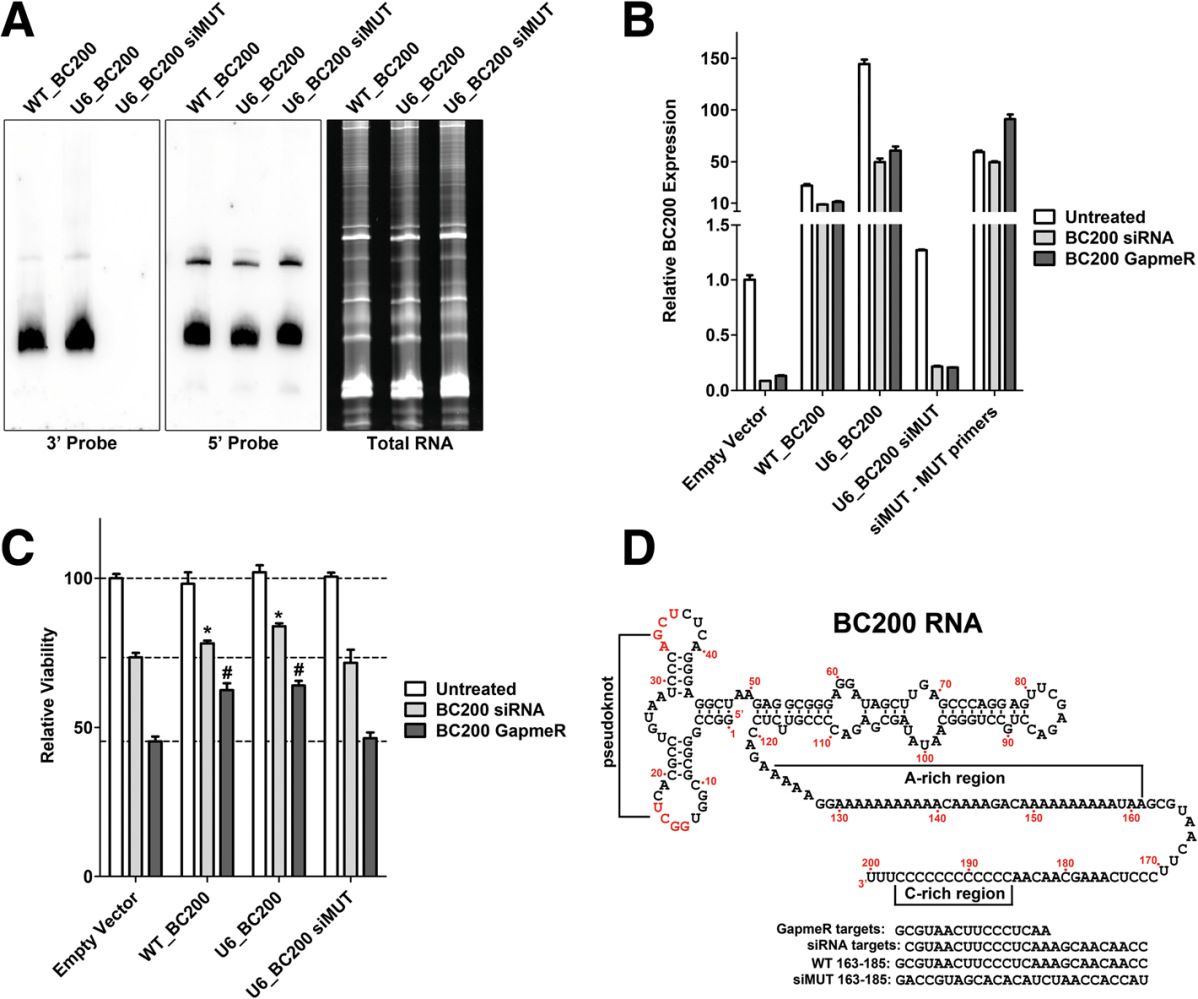
BC200 overexpression rescues the knock-down phenotype. (**a**) HeLa cells were transfected with expression vectors containing BC200 under control of the endogenous promoter (WT_BC200), BC200 under control of the U6 promoter (U6_BC200) as well as an siRNA resistant sequence mutant (U6_BC200 siMUT). BC200 was detected by denaturing TBE gel electrophoresis followed by northern blotting with DIG-labelled LNA probes targeting either the 3′ or 5′ ends of the RNA. Total RNA was detected in an identical gel run in parallel that was stained with SYBR gold. (**b**) HeLa cells were transfected with the indicated expression vectors for 24 h. 24 h post plasmid transfection cells were split an reverse transfected with BC200 siRNA or LNA GapmeR. Expression of endogenous and exogenous BC200 was assessed by qPCR with specific primers 48 h post transfection of the BC200 siRNA or LNA GapmeR. (**c**) Cell viability was measured by MTT assay 48 h post transfection of the BC200 siRNA or LNA GapmeR. Data represents the mean of 8 biological replicates +/− standard deviation. * indicates statistically significant deviation (*p* < 0.05) from Empty Vector transfected with BC200 siRNA. # indicates statistically significant deviation (*p* < 0.05) from empty vector transfected with BC200 LNA GapmeR. *P*-values were calculated by an unpaired two-tailed *t*-test. (**d**) The region of BC200 targeted by the LNA GapmeR and siRNA is shown in the context of the primary sequence and proposed secondary structure of BC200. The sequence scrambled in U6_BC200 siMUT (163-185) is shown alongside the wild-type sequence

To rule out off-target effects of the RNA interference, BC200 knock-down was rescued in HeLa cells by transfection of BC200 expression plasmids. BC200 over-expressed under control of the endogenous BC200 promoter (WT_BC200) attained levels approximately 20-fold greater than endogenous whereas expression from the U6 snRNA promoter (U6_BC200) attained expression levels approximately 150-fold greater than endogenous. In the case that the exogenous BC200 constructs would not provide sufficient expression under knock-down conditions, an siRNA resistant sequence mutant (UT_BC200 siMUT, nucleotides 163-185 scrambled) was also employed. Expression of all three constructs was confirmed by northern blot using probes specific to both the 5′ and 3′ ends of the RNA (Fig. 6a). The 3′ probe overlaps with the scrambled sequence of U6_BC200 siMUT which is therefore not detected. Plasmid transfection efficiency, as measured by a GFP reporter construct, was approximately 40-50%.

Expression of exogenous BC200 was maintained above endogenous levels 48 h post-transfection of both BC200 siRNA and LNA GapmeR (Fig. 6b). The siRNA resistant mutant did not impair knock-down of endogenous BC200 and a specific reverse primer confirmed that expression of the mutant remained relatively constant following siRNA and GapmeR transfection. Transfection of WT_BC200 and U6_BC200 significantly protected against the loss of viability observed upon BC200 siRNA and GapmeR transfection as compared to empty vector (Fig. 6c). In contrast, the siRNA resistant mutant did not differ significantly from empty vector control, suggesting that the sequence modified is likely critical for BC200 function. The sequence targeted for knock-down as well as the mutant sequence are demonstrated in Fig. 6d in the context of the full-length RNA.

In addition to rescue experiments, BC200 was overexpressed in five cell lines. BC200 overexpression had no significant impact on cell viability 72-h post transfection in all cell lines tested (Additional file 5: Figure S5a). Furthermore, overexpression of BC200 combined with serum deprivation or exposure to cytotoxic agents demonstrated no change in viability as compared to empty vector (Fig. 5b).

### BC200 expression is reduced by factors inhibiting cell proliferation

**Fig. 7:**
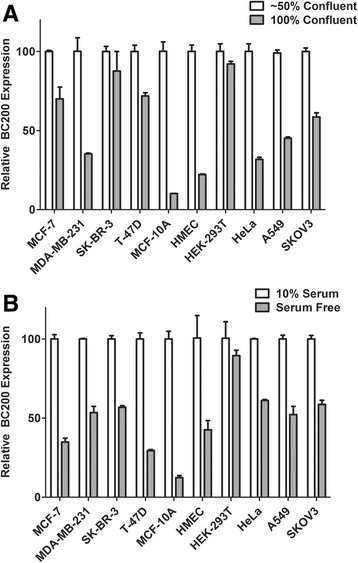
BC200 expression is reduced by confluence and serum deprivation. (**a**) Cells were plated at a concentration of 2 × 10^5^ (~50% confluent) or 5 × 10^5^ (100% confluent) and collected following 48-h culture. BC200 expression was measured by RT-qPCR and corrected to the housekeeping gene GAPDH. Data represents the mean of triplicate measurements +/− standard error. (**b**) Cells were plated at a concentration of 2 × 10^5^. Following 24-h media was changed to serum free DMEM. BC200 expression was assessed 24 h following serum deprivation by RT-qPCR as in (**a**)

**Fig. 8:**
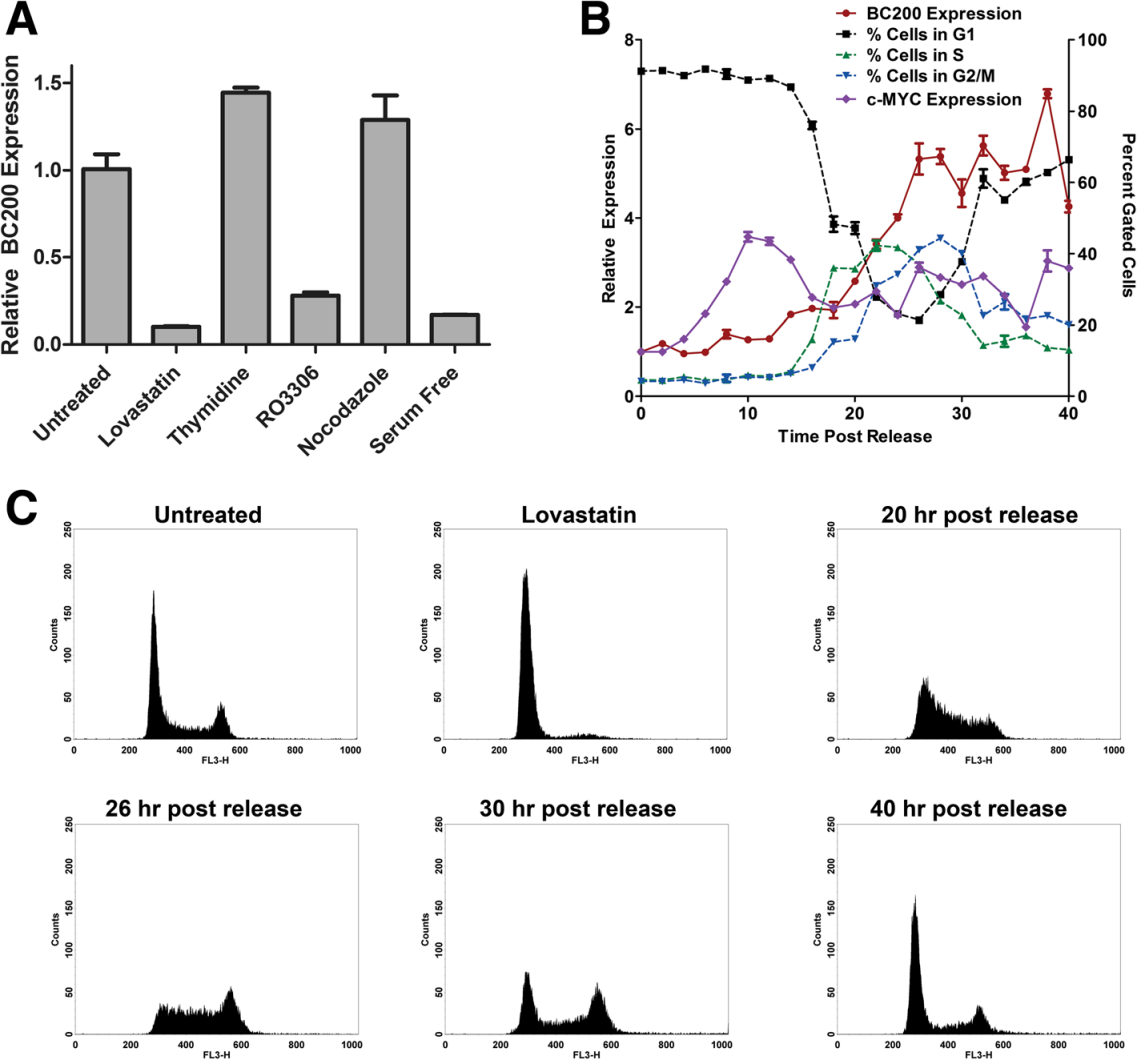
Cell cycle arrest reduces BC200 expression approximately 10-fold. (**a**) MCF-10A cells were treated with Lovastatin (40 μM), thymidine (2 mM), RO3306 (10 μM), nocodazole (0.1 μg/mL), or serum deprivation for 24 h to arrest cells in G1, S, G2, M and G1-phase respectively. BC200 expression was assessed by RT-qPCR and corrected to the housekeeping gene GAPDH. Data represents the mean of three replicates +/− standard error. Similar results were observed in HMEC cells (data not shown). (**b**) MCF-10A cells were arrested in the G1-phase by treatment with Lovastatin for 24 h. Cells were washed and released with mevalonic acid and collected every two hours to monitor BC200 and MYC expression and cell cycle phase. BC200 and MYC expression are represented on the left y-axis and the fraction of cells in each phase as determined by DNA content is represented by the right y-axis. (**c**) Histograms for selected time points demonstrate synchronous progression through the cell cycle following release from Lovastatin arrest. DNA content is monitored by propidium iodide fluorescence intensity on the x-axis (FL3-H)

We observed variations in BC200 expression that correlated with cell density at the time of collection. To investigate this observation, cells were plated such that they would be either completely confluent or approximately 50% confluent on the day of collection. With the exception of SK-BR-3 and HEK-293 T cells, BC200 expression was markedly reduced upon cell confluence (Fig. 7a). Results were most prominent in the non-tumorigenic MCF-10A cells and the primary mammary epithelial cells. To test if a reduction in BC200 expression correlated with growth inhibition, we also measured BC200 levels in cells following 24 h of serum deprivation. With the exception of HEK293T cells, BC200 levels were significantly reduced under serum-free conditions (Fig. 7b). This led us to test whether BC200 expression correlated with actively cycling cells. To test if BC200 expression is cell cycle dependent, MCF-10A cells were arrested at various stages of the cell cycle using specific chemical inhibitors [20]. Treatment of cells with Lovastatin (G1 arrest) [24, 25], and RO3306 (G2 arrest) [26] reduced BC200 expression whereas treatment with Thymidine (S-phase arrest) [20] and Nocodazole (M-phase arrest) [20] resulted in a moderate elevation in BC200 expression (Fig. 8a).

**Fig. 9:**
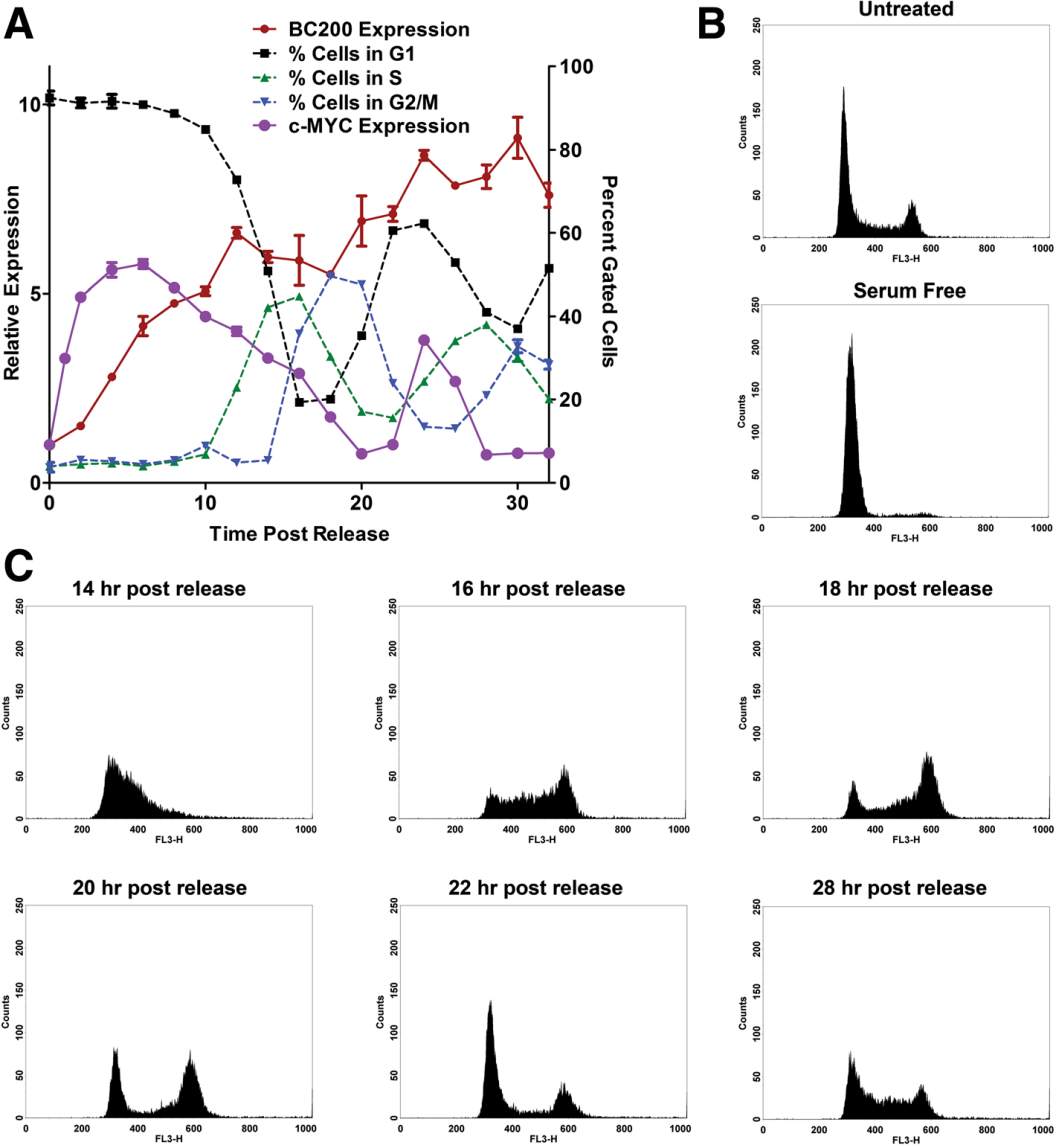
BC200 expression is regained upon growth factor reintroduction prior to exit from G1 phase. (**a**) MCF-10A cells were serum-deprived for 24 h followed by reintroduction of growth factors. Cells were collected every two hours to monitor BC200 and MYC expression by qPCR as well as cell cycle progression by flow cytometry. BC200 and MYC expression are represented on the left y-axis and the fraction of cells in each phase as determined by DNA content is represented by the right y-axis. (**b**) Histograms demonstrate the cell cycle profile of untreated and serum-deprived MCF-10A cells. (**c**) Histograms for selected time points demonstrate synchronous progression through the cell cycle following growth factor reintroduction

To determine if BC200 is periodically expressed throughout the cell cycle, MCF-10A cells were synchronized in G1 by treatment with Lovastatin for 24-h followed by release with mevalonic acid. BC200 expression began to increase at the same time as cells exited G1 (approximately 18-h post release), peaked at approximately 26-h, and remained elevated through the remainder of the experiment (Fig. 8b). Cell cycle progression was assessed by monitoring DNA content by flow cytometry (Fig. 8c). To confirm the results obtained with Lovastatin synchronization, MCF-10A cells were also synchronized in G1 by growth factor deprivation. Following 24-h of serum deprivation >90% of the cells were in G1-phase and BC200 expression was reduced approximately 10-fold (Fig. 9a, b). Synchronized cells did not begin exiting G1 phase until 10-h; however, BC200 expression began to increase within 2-h after addition of growth factors. Expression steadily increased, peaking at approximately 24 h and remaining elevated throughout the remainder of the experiment (Fig. 9a). Cell cycle progression was assessed by monitoring cellular DNA content by flow cytometry (Fig. 9b, c).

As Hu et al. reported MYC regulated transcription of BC200 in lung cancer, we also examined c-MYC expression patterns following release from synchronization [9]. While MYC expression was rapidly elevated following addition of serum (Fig. 9a), a delay was observed in MYC induction following Lovastatin release (Fig. 8b). This result was consistent with the rapid induction of BC200 observed upon introduction of serum and the delayed induction observed upon Lovastatin release. We also assessed BC200 expression following targeted MYC knock-down by siRNA. MYC knock-down in MCF-7 cells resulted in a 70% reduction in BC200 at 24 h (Additional file 6: Figure S6A, B). This data supports the notion that BC200 is also regulated by MYC in the context of breast cancer.

### Inhibition of cell cycle progression counteracts the impact of BC200 knock-down on cell viability

**Fig. 10:**
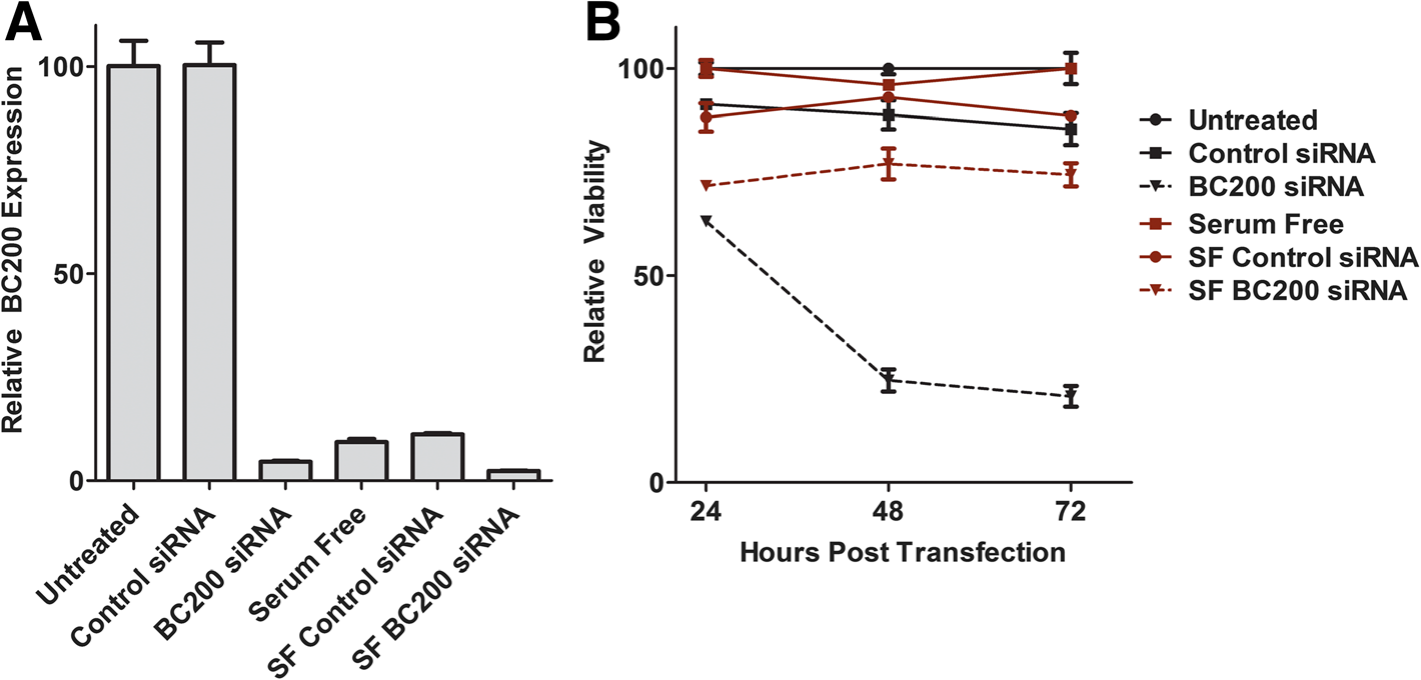
Non-dividing cells are protected from viability loss due to BC200 knock-down. (**a**) MCF-10A cells were transfected with either control or BC200 targeting siRNA. Six-hours post-transfection media was changed to either complete media or media lacking growth factors (SF). BC200 expression was assessed 24-h post transfection by RT-qPCR. (**b**) Cells treated in the same manner as in (**a**) were employed for viability measurements by MTT assay over the course of 72-h post transfection. Data represents the mean of three biological replicates +/− standard error

As BC200 expression is reduced by serum deprivation to a similar extent as siRNA knock-down, we wished to test whether serum deprived cells remained sensitive to BC200 knock-down. MCF-10A cells were left untreated or transfected with control or BC200 specific siRNA. Six-hours post siRNA transfection, culture media was replaced with either complete HuMEC media or serum-free Dulbecco’s Modified Eagle’s media (DMEM). BC200 expression was assessed 48-h post-transfection at which point BC200 siRNA resulted in >95% knockdown in both complete and serum free media (Fig. 10a). Serum deprivation alone resulted in approximately 90% reduction in BC200 expression (Fig. 10a). Cell viability was monitored by MTT assay at 24-, 48- and 72-h. While knock-down followed by serum deprivation resulted in an initial reduction, viability remained constant at about 75% relative to controls throughout the course of the experiment. In contrast, cells in complete media demonstrated an 80% reduction in viability by 72-h (Fig. 10b).

## Discussion

The study of non-coding RNAs in the regulation of gene expression is a rapidly expanding field with significant implications for human health and disease. To date over 60,000 long non-coding RNAs have been identified that account for approximately 60% of the cellular transcriptome [27]. Over 4000 lncRNAs have demonstrated aberrant expression in cancer, establishing both their relevance and complexity in understanding tumor cell biology [27, 28].

While the bulk of studies investigating normal human tissue suggest BC200 has a brain-specific expression pattern, the observation that this RNA is elevated in cancer cell lines was made in the preliminary paper describing the discovery of the RNA [1]. Since then, several reports have confirmed elevated BC200 expression in tumours of disparate origin relative to matched normal tissues [3, 7–10]. Our data confirms high expression in the brain along with elevated expression in testes, ovary, and small intestine. While elevated levels in testes and ovary has been reported prior [3, 16], to our knowledge this is the first report of elevated levels in small intestine. We also present, for the first-time, direct quantification of BC200 copy number, allowing for meaningful comparisons between expression levels that are not based on relative or only qualitative methods (northern blot, in-situ hybridization). We have also performed accurate quantification of BC200 expression in 12 cell types, demonstrating that BC200 expression is ubiquitously expressed in cultured cancer and non-tumorigenic cell lines, primary breast epithelial cells, and primary cells derived from lung. Our data conflicts with a previous report that suggested elevated levels in T47D cells [10]; however, as this study analyzed expression relative to the GAPDH mRNA and we observed significantly lower expression of GAPDH mRNA in T47D cells the absolute levels of BC200 are likely in agreement. Another point of discrepancy with the literature is our report of 8-fold greater expression in SK-OV-3 cells as compared to RNA extracted from normal human ovaries. A recent report by Wu et al. indicated an average 30-fold decrease in BC200 expression in ovarian tumor samples relative to normal controls [16]. While limited to a single cell-line model, our results are in agreement with other reports observing elevated expression of BC200 in ovarian cancer relative to normal tissue [7, 29].

BC200 expression is stated to be primarily cytoplasmic and specifically dendritic in the context of neuronal cells [3]. As we were unable to attain a signal by fluorescent in-situ hybridization (FISH) that was visibly reduced by BC200 knock-down (data not shown), and as all localization data to date relied upon hybridization methods that lacked a reliable negative control, we sought to confirm these findings by an alternative method of cell fractionation and quantitative PCR. These results confirmed the localization of BC200, as less than 2% of the total BC200 RNA was detected in the nuclear fraction in both MCF-7 and MDA-MB-231 cells.

BC200 knock-down by siRNA and LNA GapmeRs reduced viability and induced apoptosis in a broad spectrum of cell lines. These data strongly indicate that BC200 expression is critical for the viability of proliferating cultured cells. This is in agreement with a recent report indicating that targeted knock-out of BC200 by CRISPR/Cas9 reduced viability of MCF-7 cells [10]. Two additional studies employed siRNA approaches to knock-down BC200 expression in both lung [9] and ovarian cancer [16] cell lines. While knock-down in A549 cells reduced cell motility, the authors did not report a loss in cell viability or induction of apoptosis. The discrepancy with our data may be primarily due to a significantly higher knock-down efficiency under our experimental conditions (96% vs 80% in A549 cells). In ovarian cancer cell lines Wu et al. report knock-down of BC200 as having a positive impact on cell proliferation and report a protective effect against carboplatin-induced apoptosis in SK-OV-3 cells [16]. This is in stark contrast to our data in which the SK-OV-3 cell line demonstrated a > 50% decrease in cell viability and approximately 50% cell death upon knock-down with a BC00 specific siRNA or LNA GapmeR. Wu et al., reported knock-down efficiencies that were within a similar range as what we report of approximately 75% and the siRNAs used targeted a similar region of BC200. While all siRNAs targeting other regions of BC200 were ineffective at knocking down BC200, an LNA GapmeR targeting nucleotides 67-81 also reduced viability of SK-OV-3 cells (Additional file 3: Figure S3).

Loss of viability due to BC200 knock-down was attributable to a combination of growth inhibition and induction of apoptosis that was highly variable amongst the cell lines tested. While MDA-MB-231 cells demonstrated a substantial loss in viability, only 20-25% of cells were apoptotic 72-h post knock-down. This contrasts with MCF-7 and MCF-10A cells, where a similar viability loss was observed by MTT assay and the majority of cells were apoptotic at 72-h. There were also some cell-type specific discrepancies in the efficacy of the siRNA and LNA GapmeR knock-down approaches. This is exemplified in the HMEC cells in which at 72 h the LNA GapmeR did not result in a loss of viability compared to control while a 50% reduction in viability was observed with siRNA transfection. This trend was reversed in the context of HEK293T cells where the LNA GapmeR was significantly more effective than siRNA transfection. While knock-down efficiencies measured at 48-h were similar between the LNA and siRNA based approaches, it is possible that knock-down kinetics at earlier time points may play some role in this variation.

While over-expression of wild-type BC200 could partially rescue the knock-down phenotype, an siRNA resistant sequence mutant gave similar results as the empty vector control. This is suggestive that the sequence mutated is critical for BC200 function. This notion is supported by sequence conservation within this region as was previously reported by Skryabin et al. [30].

To gain insight into the mechanism of apoptosis induction following BC200 knock-down by siRNA, we assessed cleavage of the initiator caspases 2, 8 and 9 by western blot. While we observed an upregulation and cleavage of caspase 8 at approximately 48 h post siRNA transfection, cleavage of caspase 2 and 9 was not detected. This implicates the extrinsic apoptotic pathway as the mechanism by which BC200 knock-down is initiating programmed cell death. A further understanding of the specific mRNAs regulated by BC200 is the subject of current study and should yield insight into the mechanistic detail.

While BC200 knock-down had a dramatic impact on cell viability, over-expression of BC200 did not have any observable impact on cell viability or sensitivity to cytotoxic agents. These results were consistent across five cell lines tested that had different basal expression levels of BC200. This would suggest that over-saturation of BC200 has no discernable positive or negative impact on cell growth. It also suggests that if BC200 is acting as a translational repressor as has been reported [12–15, 17], the effect is limited to a subset of mRNAs whose expression is not critical for cell viability but may in fact be involved in negatively regulating cell proliferation and are already efficiently repressed by the endogenous BC200 expression.

BC200 expression was repressed as much as 10-fold upon confluence of cultured cells. This was most pronounced in the primary and non-tumorigenic breast cells and is likely due to contact inhibition, as serum deprivation and cell cycle inhibition demonstrated a similar effect. The notable exception in both cases were the HEK-293 T cells, which is intriguing in that these cells have a suspected neuronal origin [31]. Abundant expression in brain is suggestive that BC200 regulation in neuronal cells is quite distinct from that in cultured primary and tumour cells and this may be evidenced in our observations with HEK293T cells. Initial results with cell cycle stage-specific inhibitors suggested that BC200 expression may be periodic; however, following expression in cells synchronized by Lovastatin or serum deprivation revealed that although expression is greatly reduced in cells arrested in G1, levels remain relatively constant once cycling is resumed. This was most evident in the serum deprived cells where a synchronized return to G1-phase is observed without discernible decrease in BC200 expression.

A previous report demonstrates MYC-regulated BC200 expression in the context of lung cancer [9]. The expression pattern of BC200 in breast epithelial cells following release from cell cycle arrest by both Lovastatin and serum withdrawal occurs following a spike in MYC expression level. In the case of Lovastatin withdrawal, where induction of MYC expression is delayed by approximately 8 h, we see a similar delay in BC200 induction. Furthermore, we observed a significant reduction in BC200 expression following MYC knock-down by siRNA. These data support the hypothesis that BC200 expression is also regulated by MYC in the context of breast cancer.

Finally, the utility of BC200 as a cancer-cell-specific therapeutic target is bolstered by results demonstrating that growth inhibition and apoptosis induction by BC200 knock-down are limited to actively dividing cells. Knock-down of BC200 followed by serum deprivation of MCF-10A cells results in only a marginal loss of viability. Although it is likely playing a critical role in the brain, tumour targeted delivery or the use of inhibitors that are unable to cross the blood-brain barrier may present as viable future therapeutic options. As such, a further understanding of the specific functions of BC200 in both a neurological and cancer cell context is essential is pursuing this lncRNA as a drug target.

## Conclusions

This study for the first time quantitatively assesses expression of the BC200 RNA in a wide variety of normal human tissues, cancer cell lines and cultured primary cells. We have shown that, contrary to the current literature, BC200 expression is not restricted to a tumor and neuronal expression pattern but that BC200 expression outside of the nervous system is likely a hallmark of all actively proliferating cells. We have developed multiple effective means of BC200 knock-down that result in a loss of viability and induction of apoptosis in a broad spectrum of cultured cell types. Additionally, we have demonstrated for the first time that BC200 overexpression has no impact on cell viability or sensitivity to chemotherapeutic agents. Furthermore, the novel finding that BC200 expression is substantially reduced upon cell cycle arrest and rapidly induced upon resumption of proliferation supports the hypothesis that BC200 plays a critical role in cell cycle progression. Finally, the novel demonstration that BC200 inhibition is only toxic to actively proliferating cells supports the rationale of targeting this lncRNA for the treatment of a broad spectrum of tumor types.

## Additional files

Additional file 1: Figure S1. Quantitative measurements of BC200 copy number by RT-qPCR. (**a**) Denaturing TBE-Urea polyacrylamide gel of in-vitro transcribed BC200 (lane 1) as well as purified fractions collected by gel filtration (lanes 3-9). (**b**) Serial dilutions of RNA purified in (**a**) were used to generate a standard curve by RT-qPCR. Data represents the mean of four replicates +/− standard error. (TIFF 1335 kb)

Additional file 2: Figure S2. Optimization of BC200 knock-down by siRNA and LNA GapmeR transfection. (**a**) HEK293T cells were transfected with the indicated siRNAs and GapmeRs and BC200 expression was assessed 48-h post transfection by RT-qPCR. siRNA_6 and GapmeR_2 were employed for all experiments unless otherwise indicated. (**b**) GapmeR_2 and GapmeR_3 were transfected into seven different cell lines to test knock-down efficiency. Efficiency of knock-down by GapmeR_3 was greatly reduced in several cell lines tested. (TIFF 740 kb)

Additional file 3: Figure S3. BC200 GapmeR_3 reduces viability to a similar degree as GapmeR_2 in cells in which knock-down is effective. (**a**) GapmeR_3 was transfected into the indicated cell lines and viability was measured by MTT assay over the course of 72 h. Data represents the mean of six biological replicates +/− standard error. (TIFF 507 kb)

Additional file 4: Figure S4. BC200 knock-down results in cleavage of caspase 8. (**a**) MCF-7 cells were transfected with a BC200 specific siRNA and cells were harvested every 8 h through 72 h post-transfection. Cleavage of caspase 2, 8 and 9 was assessed by performing SDS/PAGE followed by western blotting with specific antibodies. Antibodies to tubulin and GAPDH were used as loading controls. (TIFF 1896 kb)

Additional file 5: Figure S5. BC200 overexpression does not impact cell viability. (**a**) Plasmids expressing BC200 under control of the endogenous (WT_BC200) or U6 (U6_BC200) promoters were transfected into the indicated cell lines. Cell viability was assessed 72-h post transfection by MTT assay. Data represents the mean of six biological replicates +/− standard error. (**b**) MDA-MB-231 cells were transfected with BC200 expressing plasmids as in (**a**) and 24-h post transfection cells were changed to serum free media or treated with 10 μM cisplatin or etoposide. Viability was measured by MTT assay and is shown relative to the mean of non-transfected cells for each experimental condition. Similar results were observed with other cell lines tested (data not shown). (TIFF 754 kb)

Additional file 6: Figure S6. MYC knock-down results in reduced BC200 expression (**a**) MCF-7 cells were transfected with a MYC specific siRNA as well as a non-targeting control siRNA. BC200 expression was evaluated following 24 h by qPCR with expression normalized to the housekeeping gene GAPDH. (**b**) MYC protein levels were monitored following siRNA transfection by western blotting with a MYC specific antibody. Blots were re-probed with an anti-tubulin antibody to control for equal loading. (TIFF 455 kb)

## Abbreviations

BCYRN1: Brain cytoplasmic RNA 1
CD: Circular dichroism
DMEM: Dulbecco’s modified Eagle’s media
DTT: Dithiothreitol
EDTA: Ethylenediaminetetraacetic acid
EMSA: Electrophoretic mobility shift assay
FISH: Fluorescent in-situ hybridization
FMRP: Fragile X mental retardation protein
hTR: Human telomerase RNA
ISH: In-situ hybridization
LNA: Locked nucleic acid
lncRNA: Long non-coding RNA
PAGE: Polyacrylamide gel electrophoresis
PBS: Phosphate buffered saline
PI: Propidium iodide
RHAU: RNA Helicase associated with AU-rich element
RT-qPCR: Real-time quantitative PCR
SSC: Saline sodium citrate
TBE: Tris-borate EDTA
