# Supplementary figures and images for: The long non-coding RNA BC200 (BCYRN1) is critical for cancer cell survival and proliferation
Source: Mol Cancer. 2017 Jun 26;16:109. doi: 10.1186/s12943-017-0679-7 (PMC5483959; doi:10.1186/s12943-017-0679-7)

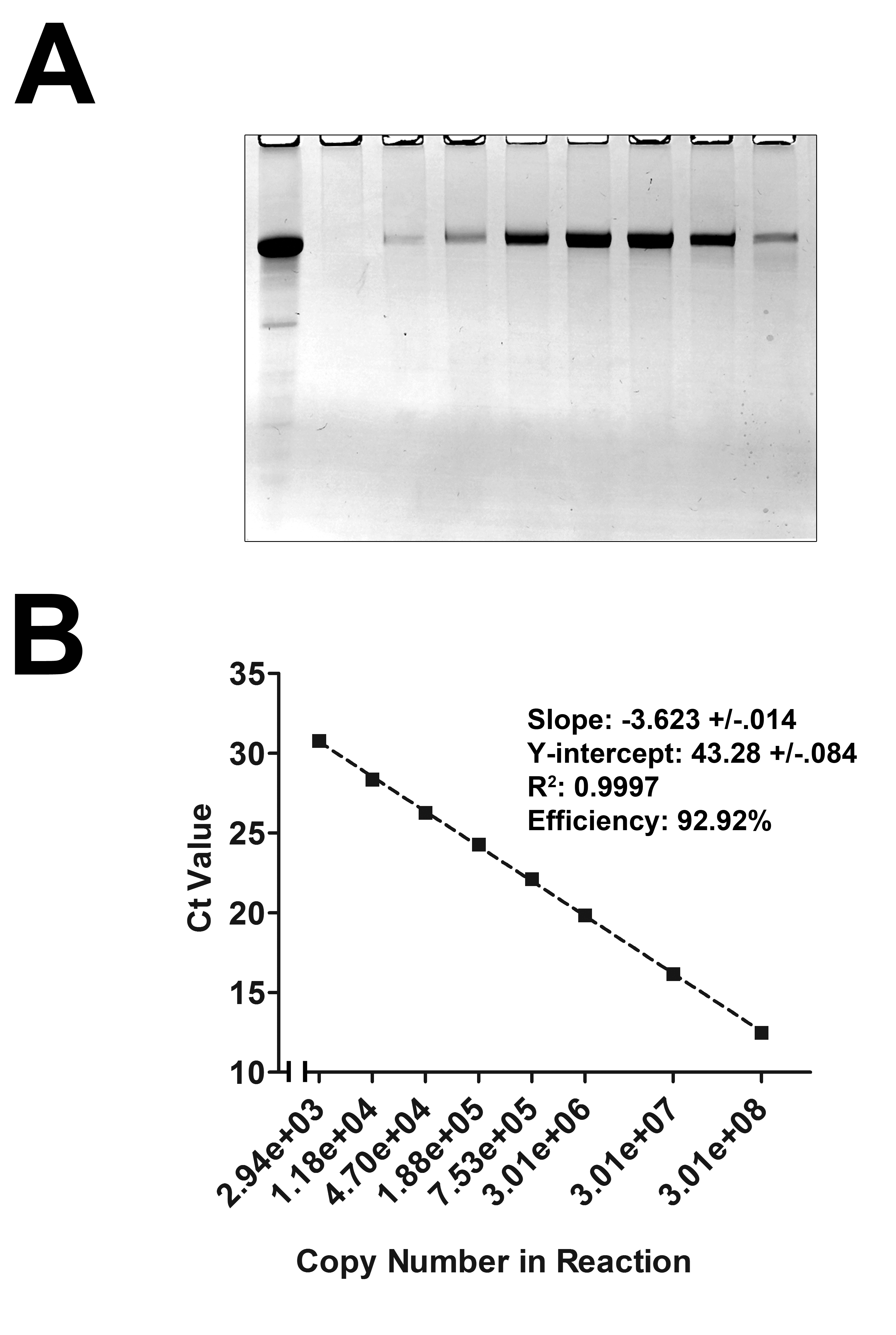

Supplement: Supplementary file 1 — Quantitative measurements of BC200 copy number by RT-qPCR. (a) Denaturing TBE-Urea polyacrylamide gel of in-vitro transcribed BC200 (lane 1) as well as purified fractions collected by gel filtration (lanes 3-9). (b) Serial dilutions of RNA purified in (a) were used to generate a standard curve by RT-qPCR. Data represents the mean of four replicates +/− standard error. (TIFF 1335 kb) [file 12943_2017_679_MOESM1_ESM.tif]

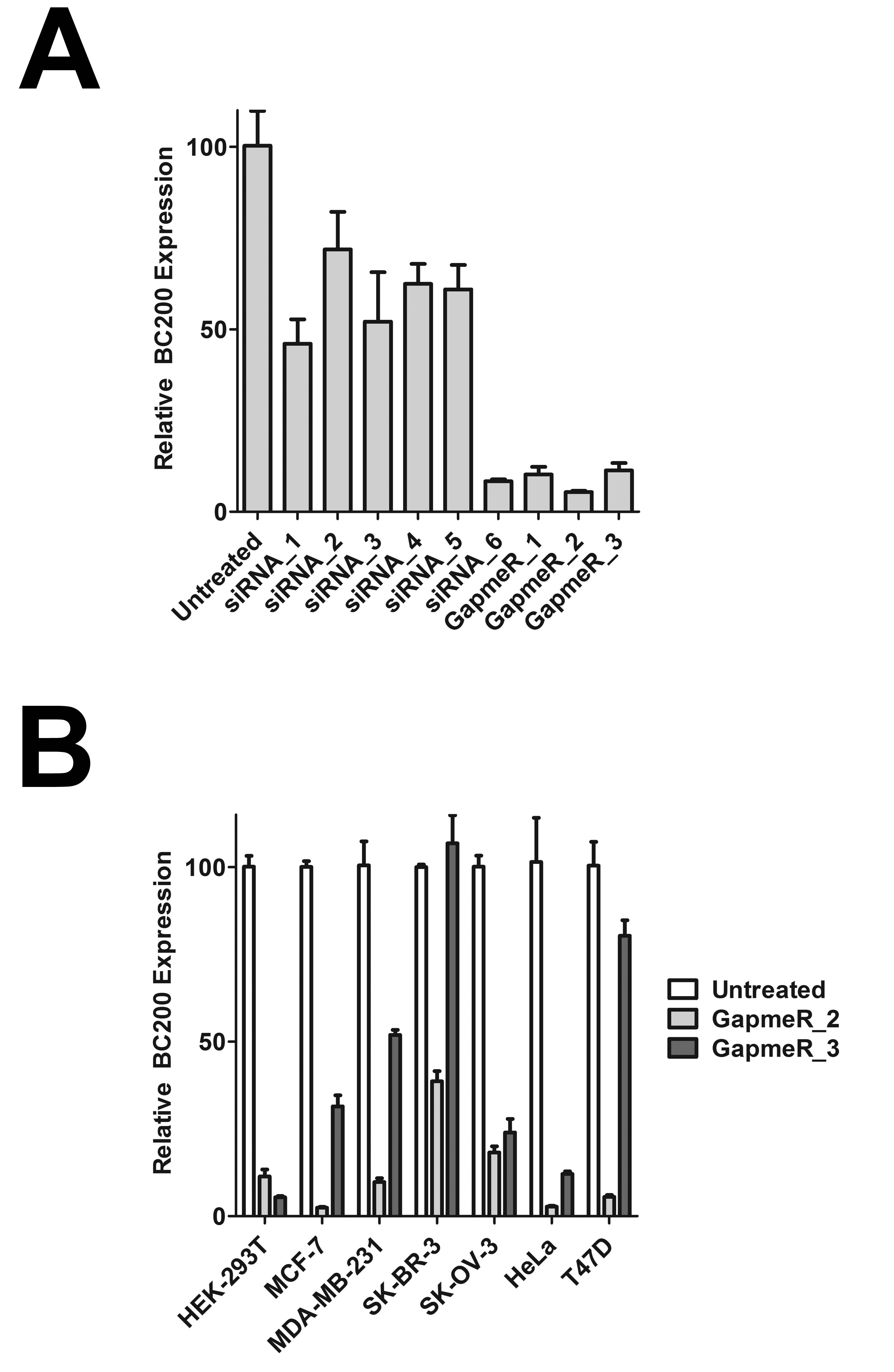

Supplement: Supplementary file 2 — Optimization of BC200 knock-down by siRNA and LNA GapmeR transfection. (a) HEK293T cells were transfected with the indicated siRNAs and GapmeRs and BC200 expression was assessed 48-h post transfection by RT-qPCR. siRNA_6 and GapmeR_2 were employed for all experiments unless otherwise indicated. (b) GapmeR_2 and GapmeR_3 were transfected into seven different cell lines to test knock-down efficiency. Efficiency of knock-down by GapmeR_3 was greatly reduced in several cell lines tested. (TIFF 740 kb) [file 12943_2017_679_MOESM2_ESM.tif]

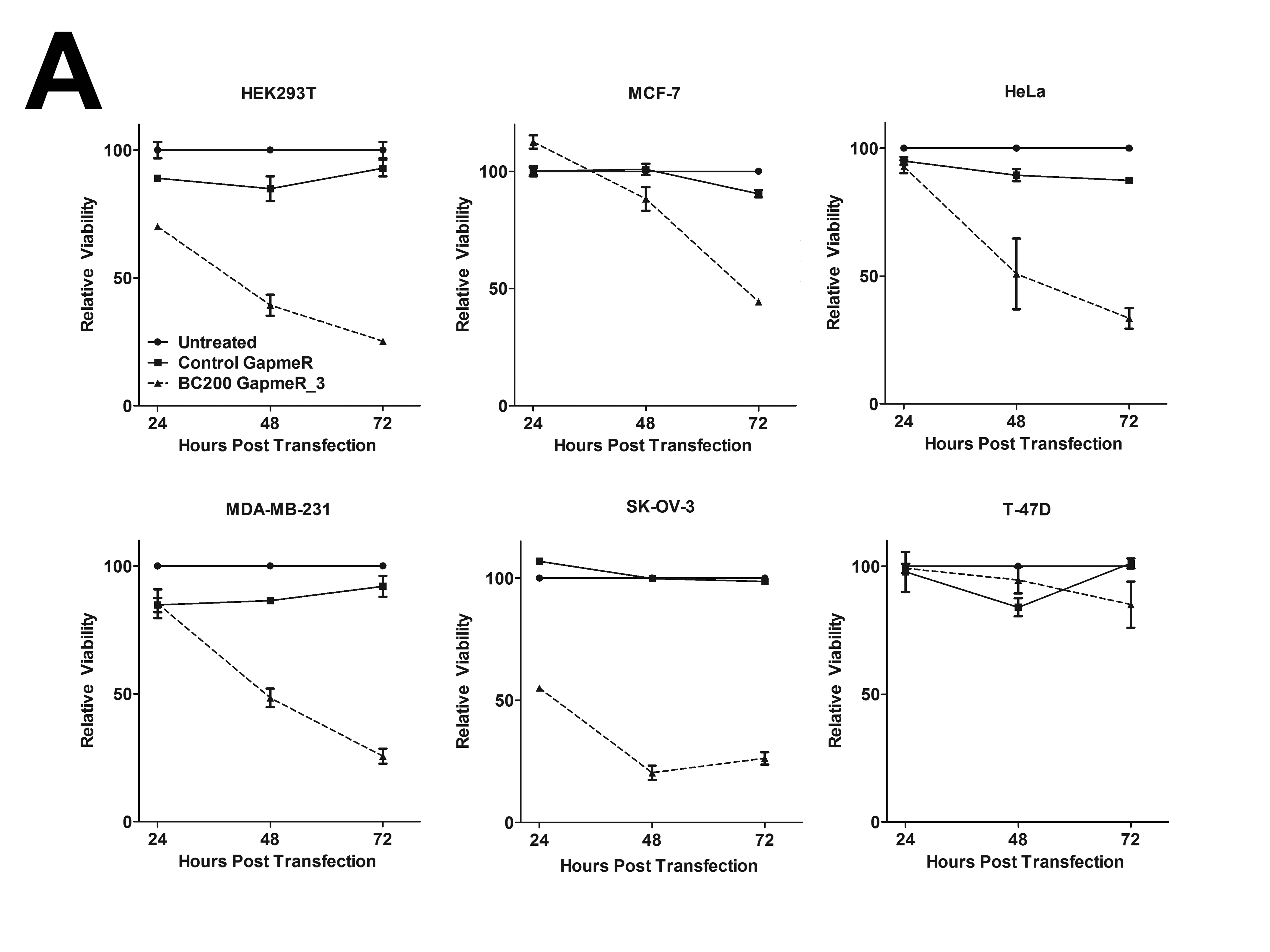

Supplement: Supplementary file 3 — BC200 GapmeR_3 reduces viability to a similar degree as GapmeR_2 in cells in which knock-down is effective. (a) GapmeR_3 was transfected into the indicated cell lines and viability was measured by MTT assay over the course of 72 h. Data represents the mean of six biological replicates +/− standard error. (TIFF 507 kb) [file 12943_2017_679_MOESM3_ESM.tif]

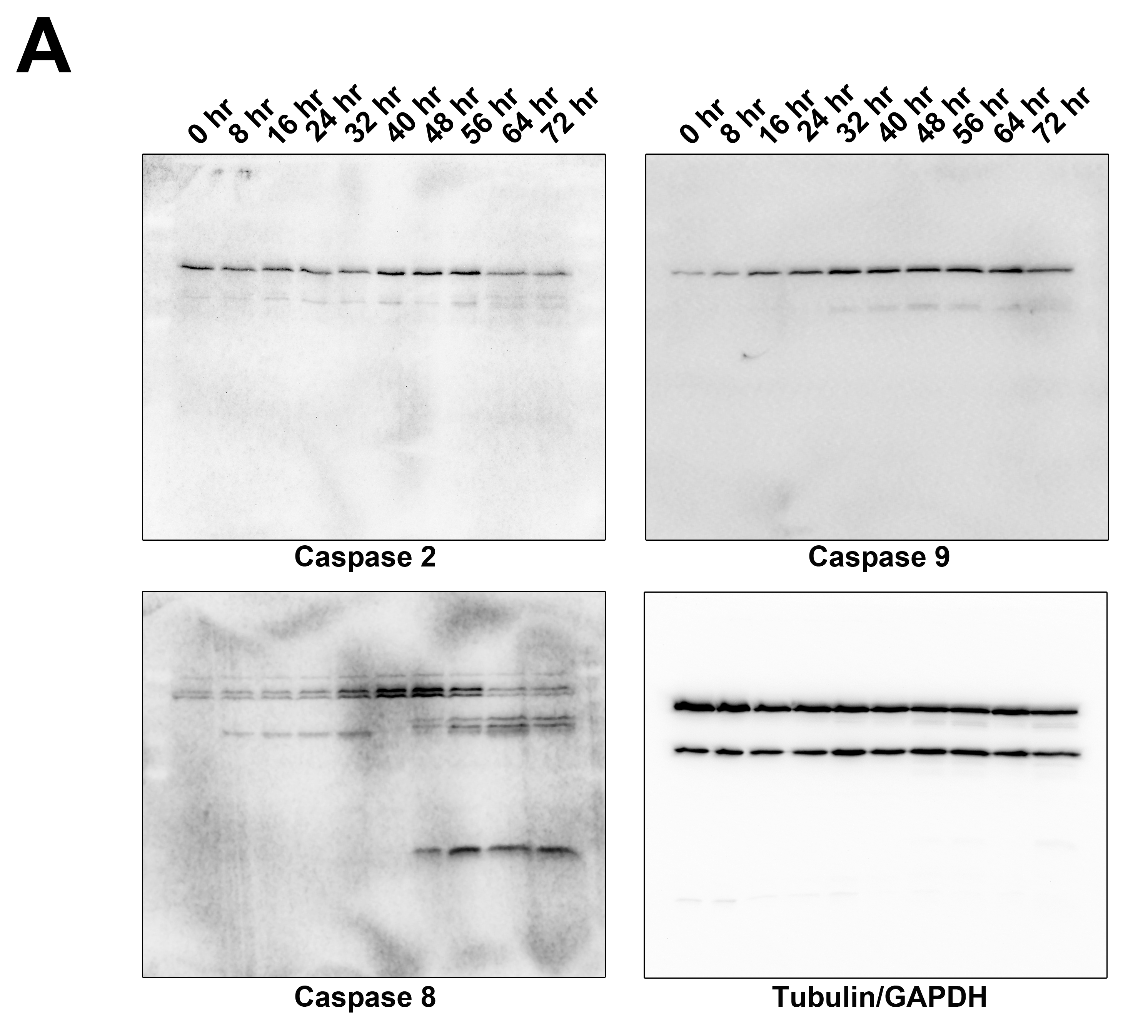

Supplement: Supplementary file 4 — BC200 knock-down results in cleavage of caspase 8. (a) MCF-7 cells were transfected with a BC200 specific siRNA and cells were harvested every 8 h through 72 h post-transfection. Cleavage of caspase 2, 8 and 9 was assessed by performing SDS/PAGE followed by western blotting with specific antibodies. Antibodies to tubulin and GAPDH were used as loading controls. (TIFF 1896 kb) [file 12943_2017_679_MOESM4_ESM.tif]

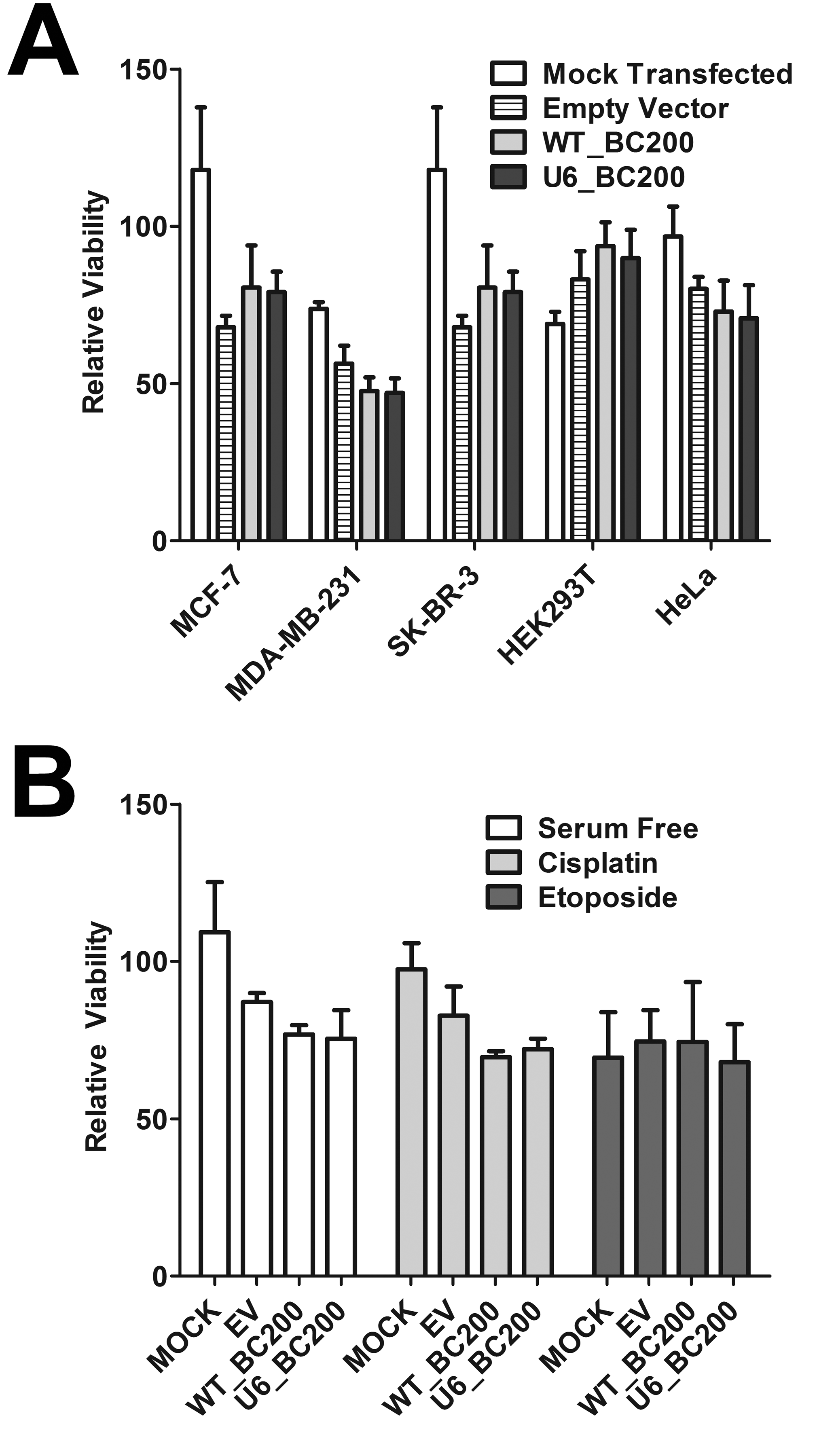

Supplement: Supplementary file 5 — BC200 overexpression does not impact cell viability. (a) Plasmids expressing BC200 under control of the endogenous (WT_BC200) or U6 (U6_BC200) promoters were transfected into the indicated cell lines. Cell viability was assessed 72-h post transfection by MTT assay. Data represents the mean of six biological replicates +/− standard error. (b) MDA-MB-231 cells were transfected with BC200 expressing plasmids as in (a) and 24-h post transfection cells were changed to serum free media or treated with 10 μM cisplatin or etoposide. Viability was measured by MTT assay and is shown relative to the mean of non-transfected cells for each experimental condition. Similar results were observed with other cell lines tested (data not shown). (TIFF 754 kb) [file 12943_2017_679_MOESM5_ESM.tif]

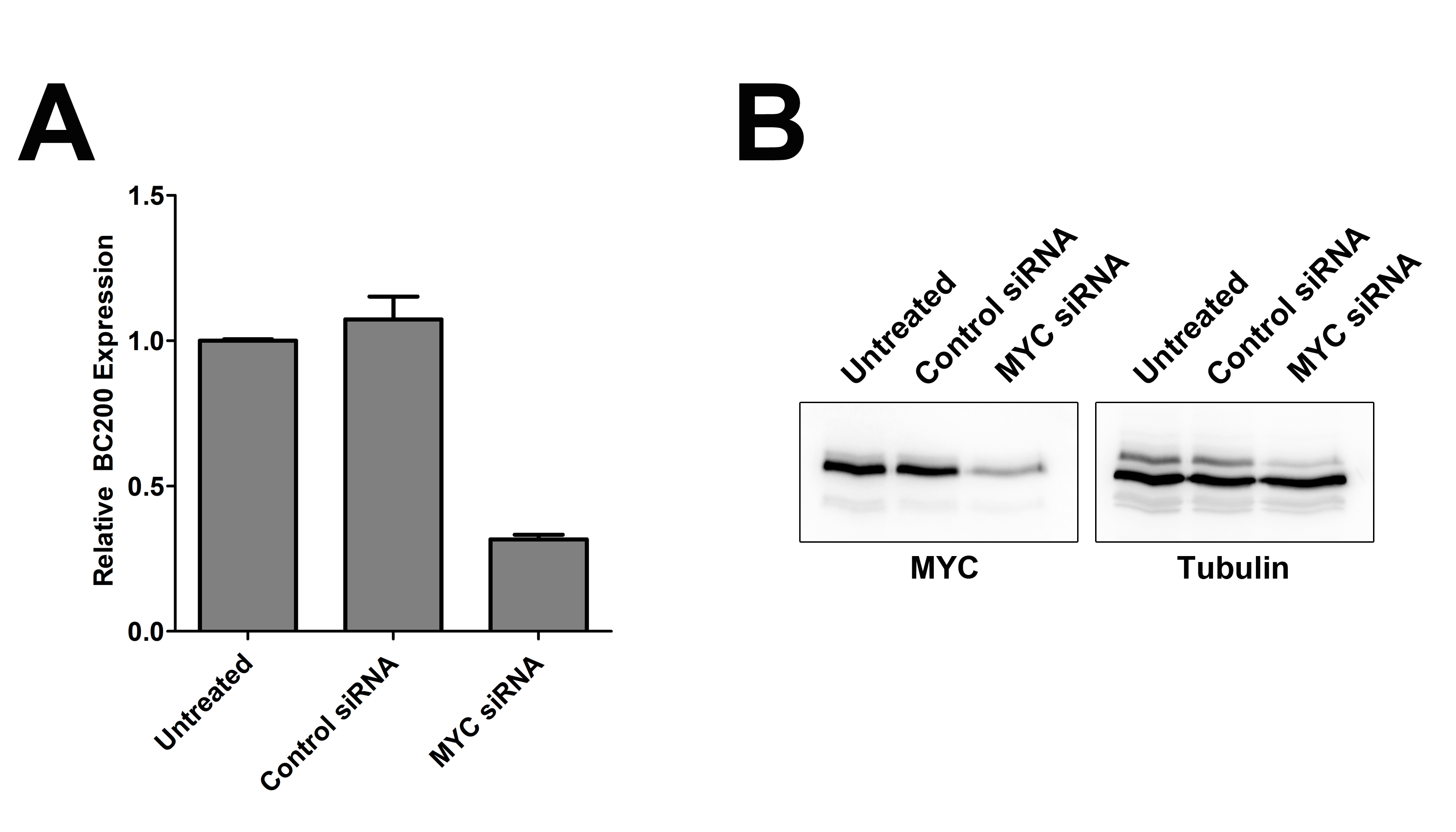

Supplement: Supplementary file 6 — MYC knock-down results in reduced BC200 expression (a) MCF-7 cells were transfected with a MYC specific siRNA as well as a non-targeting control siRNA. BC200 expression was evaluated following 24 h by qPCR with expression normalized to the housekeeping gene GAPDH. (b) MYC protein levels were monitored following siRNA transfection by western blotting with a MYC specific antibody. Blots were re-probed with an anti-tubulin antibody to control for equal loading. (TIFF 455 kb) [file 12943_2017_679_MOESM6_ESM.tif]
